# Perceptions of e-Cigarettes and Heated Tobacco Products Among Never Users of Nicotine in the European Union

**DOI:** 10.1093/ntr/ntaf168

**Published:** 2025-08-01

**Authors:** Charlotte Xin Li, Pin-Chun Wang, Ariadna Feliu, Anthony A Laverty, Cristina Martinez, Filippos T Filippidis

**Affiliations:** Department of Primary Care and Public Health, School of Public Health, Imperial College London, London, UK; European Network for Smoking and Tobacco Prevention, Brussels, Belgium; Department of Primary Care and Public Health, School of Public Health, Imperial College London, London, UK; Department of Primary Care and Public Health, School of Public Health, Imperial College London, London, UK; CIBER en Enfermedades Respiratorias (CIBERES), Madrid, Spain; Department of Primary Care and Public Health, School of Public Health, Imperial College London, London, UK; CIBER en Enfermedades Respiratorias (CIBERES), Madrid, Spain; Tobacco Control Unit, Cancer Control and Prevention Program, Institut Català d’Oncologia, L’Hospitalet de Llobregat, Barcelona, Spain; Cancer Control and Prevention Group, Institut d’Investigació Biomèdica de Bellvitge-IDIBELL, L’Hospitalet de Llobregat, Barcelona, Spain; Department of Public Health, Maternal Health and Mental Health, School of Nursing, Universitat de Barcelona, L'Hospitalet del Llobregat, Barcelona, Spain; Philip R. Lee Institute for Health Policy Studies, University of California San Francisco, San Francisco, CA, USA; Department of Primary Care and Public Health, School of Public Health, Imperial College London, London, UK

## Abstract

**Introduction:**

Emerging tobacco and nicotine products, such as e-cigarettes and heated tobacco products (HTPs), are gaining popularity, particularly among young people. This study examines the perceptions of e-cigarettes and HTPs among nicotine-naive individuals and their variations across sociodemographic subgroups.

**Methods:**

We conducted a cross-sectional analysis of the Special Eurobarometer 99.3 (May–June 2023) among never users of tobacco or nicotine products in the European Union (EU) (*n* = 13 436). We estimated the weighted prevalence of perceptions of e-cigarettes and HTPs: appeal, perceived effectiveness for smoking cessation, support for them being regulated like cigarettes, and support for keeping them out of sight in points of sale. Multi-level Poisson regression models examined associations of sociodemographic factors with these perceptions.

**Results:**

Among never users in the EU, 2.6% found e-cigarettes appealing, while 2.0% found HTPs appealing. Fifty-point eight percent and 58.8% of participants supported strict regulations and point-of-sale restrictions for these products, respectively. Younger age groups were more likely to find e-cigarettes (prevalence ratio [PR] = 1.70, for ages 15–39 vs. ≥55) and HTPs (PR = 1.88, PR = 1.54 for ages 15–24 and 25–39) appealing and view them as effective for smoking cessation. Support for regulations similar to cigarettes was higher among women, individuals with higher education, those living with children, and those without financial difficulties.

**Conclusion:**

While the appeal of these products to never users remained low overall, the study found that young people were more likely to find them appealing, posing a risk for experimentation. Meanwhile, over 50% of respondents supported stricter regulations, which could influence policy changes in this area.

**Implications:**

Although appeal of e-cigarettes and Heated Tobacco Products (HTPs) was relatively low among people who have never used e-cigarettes, HTPs, or smoking tobacco in the EU, appeal was higher among younger age groups, which are often targeted by the industry. We also found substantial support for stricter regulatory policies for e-cigarettes and HTPs across the EU. These findings might encourage governments to increase restrictions around these emerging nicotine and tobacco products.

## Introduction

The tobacco epidemic remains one of the most significant yet preventable health threats in the European Union (EU), accounting for approximately 700 000 deaths annually.^1^ Despite considerable progress in tobacco control in recent years, the prevalence of smoking remains high at 24%.^2^ Emerging nicotine and tobacco products, such as e-cigarettes and heated tobacco products (HTPs), pose public health challenges, as their popularity has grown across the EU, particularly among adolescents and young adults, some of whom have never used tobacco before.^2^ Although there is debate over whether e-cigarettes and other emerging nicotine and tobacco products can lower health risks compared to traditional cigarettes and, hence, may play a role in tobacco control, it is universally recognized that they are not risk-free and can cause harm to individuals who have never smoked.^3^ Products containing nicotine are highly addictive, and even experienced users can develop dependency levels similar to those of traditional smokers.^3–5^ Moreover, evidence suggests that never smokers who have experimented or used e-cigarettes have higher odds of transitioning to conventional tobacco smoking.^6–8^

Due to the rising popularity of HTPs, the EU activated a clause of the Tobacco Products Directive (TPD) in November 2022 to introduce a ban on characterizing flavors and packaging that alters the sensory experience.^9^ However, the most recent EU-level regulations regarding e-cigarettes date back to 2016, when measures were introduced to limit maximum nicotine concentrations, regulate product volume, and enforce child-resistant packaging and purity standards.^10^

Existing literature on perceptions of e-cigarettes and HTPs has primarily focused on current and former smokers, examining factors such as the products’ appeal and their effectiveness in aiding smoking cessation.^11–13^ These studies suggest that while e-cigarettes and HTPs are often regarded as potential cessation tools, their perceived effectiveness is lower among younger individuals, those with lower educational levels, and the unemployed.^11–13^ Moreover, younger people tend to find these products appealing, which underscores the potential vulnerability of this demographic, particularly among socioeconomically disadvantaged groups.^3^

However, a gap exists in research regarding the perceptions of these emerging tobacco and nicotine products among never users of tobacco or nicotine. Although this group has not yet used these products, they are often targeted by marketing strategies and societal trends.^14^^,^^15^ Previous studies indicate that younger individuals, particularly women, are more likely to be attracted to these products.^16^^,^^17^ The increasing influence of online retailers, which market e-cigarettes and HTPs as socially acceptable and less harmful alternatives to traditional cigarettes, raises concerns about their potential impact on never users.^18^^,^^19^ Additionally, visual exposure to these products has been shown to increase cravings and may encourage product initiation among never users.^20^ These findings highlight the potential impact of emerging tobacco and nicotine products on nicotine-naive individuals and call for further exploration of how they view e-cigarettes and HTPs.

Given these concerns, a deeper understanding of never users’ perceptions of e-cigarettes and HTPs is essential for shaping effective public health policies. To inform future regulations on e-cigarettes and HTPs within the EU, this study examines the perceptions of these products among never users of smoking tobacco, e-cigarettes, or HTPs. The study focuses on four key aspects: appeal, perceived effectiveness for smoking cessation, views on whether they should be regulated like cigarettes, and support for stricter point-of-sale restrictions. Furthermore, it explores how these perceptions vary across different sociodemographic subgroups and EU Member States (MSs). Ahead of the anticipated revision of the TPD and in light of the adoption of new recommendations for smoke- and aerosol-free environments by the Council of the EU,^21^ it is crucial to examine public perceptions of emerging tobacco and nicotine products among never users to guide future regulations.

## Methods

### Data Source

We utilized data from the Special Eurobarometer 99.3, which was conducted from May 10 to June 5, 2023. The Eurobarometer survey employed a multi-stage sampling approach. Initially, primary sampling units were selected from each country region in proportion to population size. Then, households were selected through standard “random route” methods, with one participant randomly chosen from each household based on its size to complete the interview and provide self-reported data in the relevant national language.^2^ We present questions in the English language, although these were translated into the relevant languages through a standardized translation procedure. To ensure the representativeness of the sample, the dataset was weighted according to age, sex, and residential area.

This survey gathered responses from 26 358 individuals aged 15 years and above, residing in each of the 27 EU MSs. Among those, we analyzed responses from individuals who had never used tobacco, e-cigarettes, or HTPs. Specifically, we only analyzed participants who responded “I have never smoked” in the question “Regarding smoking cigarettes, cigars, cigarillos, or a pipe, which of the following applies to you?” and “I have never used it” to the question “Thinking about the following products, which of the following applies to you? E-cigarettes/Heated tobacco products.” As such, our analytical sample size was 13 436 (Suppl. Table 1).

### Measures

#### Perceptions of Appeal

All participants were asked two separate questions: “Do you find e-cigarettes appealing?” and “Do you find HTPs appealing?” Responses to each question were then recoded as binary categories: “yes” or “no/don’t know.”

### Effective Aid for Smoking Cessation

Participants were asked, “Do you think the use of e-cigarettes helps tobacco smokers to quit?” and “Do you think the use of HTPs helps tobacco smokers to quit?” Responses were coded as “yes” or “no/don’t know.”

### Regulated as Strictly as Cigarettes

Respondents were asked the following questions regarding regulation: “Do you think e-cigarettes should be regulated as strictly as cigarettes?” and “Do you think HTPs should be regulated as strictly as cigarettes?” Responses were coded as “yes” or “no/don’t know.”

### Support for Stricter Point-of-Sale Restrictions

Respondents were also asked: “Would you be in favor of keeping e-cigarettes out of sight in shops or points of sale?” and “Would you be in favor of keeping HTPs out of sight in shops or points of sale?” Responses were coded as “in favor” or “not in favor/don’t know.”

### Covariates

The survey collected self-reported data on gender (male, female). Twenty-two responses “none of the above,” “non-binary,” “do not identify as male/female,” or “prefer not to say” were excluded and treated as missing values due to their low frequency. Sociodemographic data were also collected on age (15–24, 25–39, 40–54, ≥55 years), difficulties in paying bills at the end of the month over the past year (almost never/never, from time to time/most of the time), community type (rural, urban), age at which participants stopped full-time education (0–15, 16–19, ≥20, still studying), living with children (no, yes), and employment status (employed, unemployed, students/house persons/retired).

### Statistical Analysis

This study analyzed four perceptions of e-cigarettes and HTPs among never users of smoking tobacco, e-cigarettes, or HTPs, with a total of eight outcome variables. We first estimated the weighted prevalence of these perceptions for e-cigarettes and HTPs separately across the 27 EU MSs. Official weights provided in the Eurobarometer dataset were utilized.

We performed multi-level Poisson regression analyses for each of the eight outcome variables, accounting for clustering of observations within EU MSs. All models were adjusted for gender, age, difficulties in paying bills, community type, age when stopped full-time education, living with children, and employment status. Statistical analyses were conducted using Stata 17, and observations with missing values were excluded from the analysis. Missing values for each variable are shown in Supplementary Table 2. Significance level of the statistical analysis was defined as *p* < .05, and effect estimates are presented with 95% confidence intervals (CIs); intervals not spanning 1 denote statistical significance. Given that the Eurobarometer data are open-access and anonymized, ethical approval from an institutional review board was not required.

## Results

### Perceptions of e-Cigarettes Across 27 EU MSs

A total of 2.6% (95% CI: 2.2 to 3.1) of never users of tobacco, e-cigarettes, or HTPs across the EU found e-cigarettes appealing. The highest perception of appeal was observed in Malta at 10.8% (7.3 to 15.8), while the lowest was in Croatia at 0.5% (0.2 to 1.5). Overall, 11.2% (10.4 to 12.1) of never users in the EU believed that e-cigarettes help tobacco smokers quit, with Latvia having the lowest percentage at 3.3% (1.9 to 5.6) and Ireland the highest at 22.1% (18.7 to 25.9). Regarding regulation, 58.4% (57.0 to 59.7) believed that e-cigarettes should be regulated as strictly as cigarettes, with support ranging from 34.4% (30.4 to 38.6) in Romania to 85.1% (81.6 to 88.0) in Slovenia. Additionally, 50.8% (49.4 to 52.1) of EU never users supported keeping e-cigarettes out of sight in shops or points of sale, with Italy having the lowest support at 27.4% (23.8 to 31.2) and Estonia the highest at 84.1% (80.4 to 87.2) (Table 1).

**Table 1:** Prevalence of Perceptions Related to E-Cigarettes Among Never Users of Tobacco, E-cigarettes, or HTPs Across 27 EU Member States

Country	Appealing	Effective aid for smoking cessation	Regulate as strictly as cigarettes	Stricter point-of-sale advertising restrictions
	Weighted% with 95% confidence interval			
Austria	1.3% (0.5% to 3.4%)	5.7% (3.7% to 8.6%)	57.7% (52.4% to 62.8%)	45.2% (40.0% to 50.6%)
Belgium	4.2% (2.7% to 6.5%)	19.1% (15.3% to 23.5%)	69.8% (65.1% to 74.2%)	70.0% (65.3% to 74.4%)
Bulgaria	6.7% (4.6% to 9.6%)	7.8% (5.6% to 11.0%)	53.8% (48.9% to 58.6%)	45.2% (40.4% to 50.1%)
Croatia	0.5% (0.2% to 1.5%)	4.3% (2.7% to 6.9%)	66.2% (61.2% to 70.9%)	70.4% (65.5% to 74.9%)
Cyprus Republic	6.5% (3.6% to 11.4%)	6.5% (3.8% to 10.7%)	68.2% (61.5% to 74.2%)	50.9% (44.2% to 57.5%)
Czech Republic	1.8% (0.9% to 3.4%)	16.4% (13.2% to 20.3%)	48.6% (43.7% to 53.5%)	41.3% (36.6% to 46.3%)
Denmark	0.8% (0.2% to 2.8%)	21.8% (17.7% to 26.6%)	71.0% (66.2% to 75.5%)	74.8% (70.1% to 79.1%)
Estonia	7.2% (5.1% to 10.0%)	6.9% (4.8% to 9.7%)	78.7% (74.6% to 82.3%)	84.1% (80.4% to 87.2%)
Finland	1.1% (0.4% to 2.7%)	14.5% (11.1% to 18.8%)	77.3% (72.3% to 81.6%)	81.2% (76.3% to 85.3%)
France	1.3% (0.6% to 3.0%)	18.4% (14.7% to 22.9%)	61.7% (56.7% to 66.4%)	56.2% (51.1% to 61.1%)
Germany	1.9% (1.1% to 3.3%)	9.0% (7.0% to 11.4%)	61.4% (57.7% to 65.0%)	52.4% (48.6% to 56.1%)
Greece	2.8% (1.6% to 5.1%)	14.6% (11.4% to 18.5%)	73.0% (68.5% to 77.1%)	44.7% (39.9% to 49.6%)
Hungary	3.0% (1.8% to 5.1%)	8.6% (6.5% to 11.4%)	49.8% (45.6% to 54.0%)	46.6% (41.5% to 49.8%)
Ireland	0.9% (0.4% to 2.1%)	22.1% (18.7% to 25.9%)	73.4% (69.4% to 77.1%)	71.2% (67.1% to 75.0%)
Italy	2.2% (1.3% to 3.9%)	9.2% (7.1% to 11.9%)	48.1% (43.9% to 52.2%)	27.4% (23.8% to 31.2%)
Latvia	0.6% (0.2% to 2.0%)	3.3% (1.9% to 5.6%)	61.9% (57.2% to 66.5%)	46.2% (41.5% to 50.9%)
Lithuania	1.1% (0.5% to 2.8%)	7.1% (4.8% to 10.2%)	80.8% (76.8% to 84.3%)	80.7% (76.5% to 84.3%)
Luxembourg	2.8% (1.4% to 5.6%)	16.8% (12.1% to 22.7%)	72.4% (66.2% to 77.8%)	61.1% (54.3% to 67.4%)
Malta	10.8% (7.3% to 15.8%)	12.5% (8.5% to 18.0%)	78.5% (72.4% to 83.6%)	66.9% (60.0% to 73.2%)
Netherlands	1.1% (0.4% to 2.7%)	16.5% (13.4% to 20.2%)	78.5% (73.9% to 82.5%)	80.3% (75.7% to 84.3%)
Poland	4.9% (3.1% to 7.8%)	5.1% (3.5% to 7.4%)	41.3% (36.9% to 45.9%)	39.2% (34.8% to 43.8%)
Portugal	2.2% (1.3% to 3.7%)	6.2% (4.5% to 8.5%)	64.5% (60.8% to 68.1%)	61.4% (57.7% to 65.1%)
Romania	10.2% (8.0% to 13.1%)	10.8% (8.5% to 13.6%)	34.4% (30.4% to 38.6%)	35.7% (31.7% to 40.0%)
Slovakia	5.4% (3.6% to 8.1%)	8.3% (6.0% to 11.3%)	61.2% (56.7% to 65.6%)	55.0% (50.4% to 59.6%)
Slovenia	1.4% (0.7% to 3.0%)	8.4% (6.1% to 11.4%)	85.1% (81.6% to 88.0%)	71.3% (66.9% to 75.2%)
Spain	1.5% (0.7% to 3.2%)	10.4% (8.0% to 13.5%)	65.7% (61.5% to 69.7%)	59.9% (55.6% to 64.1%)
Sweden	1.4% (0.5% to 4.3%)	14.7% (11.1% to 19.1%)	76.5% (71.1% to 81.2%)	75.5% (69.9% to 80.3%)
EU	2.6% (2.2% to 3.1%)	11.2% (10.4% to 12.1%)	58.4% (57.0% to 59.7%)	50.8% (49.4% to 52.1%)

Appealing: Do you find e-cigarettes appealing?

Effective aid for smoking cessation: Do you think that the use of e-cigarettes helps tobacco smokers quit?

Regulate as strictly as cigarettes: Do you think that e-cigarettes should be regulated as strictly as cigarettes?

Stricter point-of-sale advertising restrictions: Would you be in favor of keeping e-cigarettes out of sight in shops or points of sale?

In the regression results (Table 2), women were less likely than men to view e-cigarettes as effective for smoking cessation (PR = 0.84, 0.75 to 0.95) and borderline less likely to find them appealing (PR = 0.80, 0.63 to 1.01). Compared to those aged ≥55 years, younger age groups (15–24 and 25–39 years) were more likely to find e-cigarettes appealing (PR = 1.70, 1.12 to 2.59 and PR = 1.70, 1.19 to 2.43, respectively) and to perceive them as effective for smoking cessation (PR = 1.81, 1.36 to 2.40 and PR = 1.38, 1.15 to 1.65, respectively). Individuals experiencing financial difficulties were less likely to support regulating e-cigarettes as strictly as cigarettes (PR = 0.88, 0.84 to 0.92) and enforcing strict point-of-sale restrictions (PR = 0.92, 0.87 to 0.98), compared to those with minimal or no financial difficulties. In contrast, individuals with higher education levels (completed education at age 20 or older) were more likely to support both measures (PR = 1.16, 1.08 to 1.24 and PR = 1.15, 1.07 to 1.22, respectively) compared to those who completed their education between ages 0 and 15. Additionally, individuals living with children were more likely to support regulating e-cigarettes as strictly as cigarettes and keeping them out of sight in points of sale compared to those living without children (PR = 1.05, 1.01 to 1.10 for both). Compared to those employed, students, homemakers, and retirees also expressed greater support for these measures (PR = 1.10, 1.05 to 1.15 and PR = 1.08, 1.02 to 1.14, respectively) (Table 2).

**Table 2:** Multi-level Poisson Regression Estimating Associations Between Sociodemographic Factors and Perceptions Related to E-Cigarettes Among Never Users of Tobacco, e-Cigarettes, or HTPs Across the EU

Variables	Appealing	Effective aid for smoking cessation	Regulate as strictly as cigarettes	Stricter point-of-sale advertising restrictions
	Prevalence ratio (95% confidence interval)			
Gender				
Male (Ref.)	1	1	1	1
Female	0.80 (0.63 to 1.01)	0.84 (0.75 to 0.95)	1.03 (1.00 to 1.07)	1.04 (1.00 to 1.08)
Age (years)				
55+ (Ref.)	1	1	1	1
15–24	1.70 (1.12 to 2.59)	1.81 (1.36 to 2.40)	0.95 (0.86 to 1.04)	0.96 (0.87 to 1.06)
25–39	1.70 (1.19 to 2.43)	1.38 (1.15 to 1.65)	0.98 (0.94 to 1.03)	0.97 (0.92 to 1.03)
40–54	1.04 (0.74 to 1.45)	1.16 (0.97 to 1.38)	1.01 (0.98 to 1.05)	0.98 (0.94 to 1.02)
Difficulty paying bills				
Almost never/never (Ref.)	1	1	1	1
From time to time/most of the time	1.32 (0.98 to 1.77)	1.13 (1.00 to 1.29)	0.88 (0.84 to 0.92)	0.92 (0.87 to 0.98)
Community type				
Rural (Ref.)	1	1	1	1
Urban	1.06 (0.81 to 1.38)	1.12 (0.95 to 1.32)	1.01 (0.96 to 1.07)	1.01 (0.95 to 1.07)
Education (age at completion)				
0–15 years (Ref.)	1	1	1	1
16–19 years	1.27 (0.82 to 1.95)	1.10 (0.85 to 1.42)	1.06 (0.98 to 1.15)	1.05 (0.98 to 1.11)
≥20 years	0.97 (0.60 to 1.56)	1.22 (0.92 to 1.62)	1.16 (1.08 to 1.24)	1.15 (1.07 to 1.22)
Still studying	1.79 (0.82 to 3.93)	1.42 (0.90 to 2.26)	1.11 (1.00 to 1.24)	1.04 (0.93 to 1.17)
Living with children				
No (Ref.)	1	1	1	1
Yes	1.00 (0.80 to 1.25)	1.10 (0.97 to 1.24)	1.05 (1.01 to 1.10)	1.05 (1.01 to 1.10)
Employment status				
Employed (Ref.)	1	1	1	1
Unemployed	0.96 (0.56 to 1.63)	1.31 (0.97 to 1.76)	1.06 (0.98 to 1.15)	1.03 (0.95 to 1.12)
Students/house persons/retired	0.76 (0.48 to 1.21)	0.74 (0.63 to 0.86)	1.10 (1.05 to 1.15)	1.08 (1.02 to 1.14)

Appealing: Do you find e-cigarettes appealing?

Effective aid for smoking cessation: Do you think that the use of e-cigarettes helps tobacco smokers quit?

Regulate as strictly as cigarettes: Do you think that e-cigarettes should be regulated as strictly as cigarettes?

Stricter point-of-sale advertising restrictions: Would you be in favor of keeping e-cigarettes out of sight in shops or points of sale?

### Perceptions of HTPs Across 27 EU MSs

Across the EU, 2.0% (1.6 to 2.4) of never users of tobacco, e-cigarette, or HTPs found HTPs appealing, with the lowest prevalence in Spain at 0.0% (0.0 to 0.0) and the highest in Romania at 11.5% (9.1 to 14.4). Among EU never users, 7.1% (6.5 to 7.9) believed that HTPs help tobacco smokers quit, with the lowest prevalence in Latvia at 1.5% (0.6 to 3.3) and the highest in Ireland at 16.6% (13.6 to 20.1). A total of 58.8% (57.4 to 60.1) of the respondents thought HTPs should be regulated as strictly as cigarettes, with the highest support in Slovenia at 85.0% (81.5 to 87.9) and the lowest in Romania at 37.3% (33.2 to 41.5). Additionally, across the EU, 51.1% (49.7 to 52.4) supported keeping HTPs out of sight in points of sale, with the highest support observed in Estonia at 84.1% (80.4 to 87.2) and the lowest in Italy at 27.3% (23.8 to 31.2) (Table 3).

**Table 3:** Prevalence of Perceptions Related to HTPs Among Never Users of Tobacco, e-Cigarettes, or HTPs Across 27 EU Member States

Country	Appealing	Effective aid for smoking cessation	Regulate as strictly as cigarettes	Stricter point-of-sale advertising restrictions
	Weighted% with 95% confidence interval			
Austria	1.2% (0.5% to 3.2%)	4.3% (2.6% to 7.1%)	58.9% (53.6% to 64.0%)	45.5% (40.2% to 50.9%)
Belgium	2.4% (0.5% to 1.2%)	6.3% (4.2% to 9.4%)	69.1% (64.3% to 73.6%)	69.8% (65.1% to 74.1%)
Bulgaria	5.7% (3.8% to 8.4%)	7.1% (5.0% to 10.2%)	54.1% (49.2% to 58.9%)	44.9% (40.2% to 49.8%)
Croatia	0.7% (0.3% to 2.0%)	4.4% (2.8% to 7.0%)	66.4% (61.4% to 71.0%)	70.5% (65.6% to 75.0%)
Cyprus Republic	6.2% (3.4% to 11.1%)	6.6% (3.9% to 10.9%)	68.2% (61.5% to 74.2%)	50.9% (44.2% to 57.5%)
Czech Republic	0.6% (0.2% to 1.7%)	12.4% (9.7% to 15.8%)	49.1% (44.2% to 54.0%)	40.4% (35.6% to 45.4%)
Denmark	0.6% (0.1% to 4.4%)	11.7% (8.7% to 15.7%)	71.3% (66.5% to 75.7%)	75.5% (70.8% to 79.7%)
Estonia	4.2% (2.6% to 6.5%)	5.5% (3.7% to 8.2%)	77.8% (73.7% to 81.5%)	84.1% (80.4% to 87.2%)
Finland	0.1% (0.0% to 0.8%)	13.1% (9.8% to 17.3%)	77.7% (72.7% to 82.0%)	81.9% (77.1% to 85.8%)
France	0.3% (0.0% to 2.2%)	4.6% (2.5% to 8.3%)	60.6% (55.6% to 65.4%)	56.3% (51.3% to 61.2%)
Germany	1.4% (0.7% to 2.7%)	5.3% (3.8% to 7.3%)	61.7% (58.0% to 65.3%)	52.4% (48.6% to 56.1%)
Greece	2.8% (1.6% to 5.1%)	14.7% (11.6% to 18.6%)	74.2% (69.7% to 78.2%)	44.4% (39.7% to 49.3%)
Hungary	2.3% (1.3% to 4.0%)	6.6% (4.7% to 9.1%)	50.4% (46.2% to 54.6%)	45.8% (41.7% to 50.0%)
Ireland	0.3% (0.1% to 1.3%)	16.6% (13.6% to 20.1%)	72.6% (68.6% to 76.3%)	70.7% (66.6% to 74.6%)
Italy	2.2% (1.3% to 3.8%)	8.7% (6.6% to 11.4%)	48.5% (44.3% to 52.6%)	27.3% (23.8% to 31.2%)
Latvia	0.9% (0.3% to 2.4%)	1.5% (0.6% to 3.3%)	61.5% (56.8% to 66.0%)	45.8% (41.2% to 50.5%)
Lithuania	0.2% (0.0% to 1.3%)	5.7% (3.7% to 8.7%)	81.4% (77.3% to 84.8%)	80.0% (75.8% to 83.7%)
Luxembourg	1.8% (0.7% to 4.5%)	11.0% (7.2% to 16.3%)	71.5% (65.2% to 77.0%)	62.4% (55.7% to 68.6%)
Malta	2.6% (1.2% to 5.7%)	6.0% (3.3% to 10.5%)	77.7% (71.4% to 82.9%)	66.9% (59.9% to 73.2%)
Netherlands	0.5% (0.1% to 2.0%)	6.4% (4.6% to 8.9%)	80.8% (76.6% to 84.4%)	81.0% (76.4% to 84.8%)
Poland	3.9% (2.3% to 6.7%)	5.0% (3.3% to 7.4%)	41.3% (36.9% to 45.9%)	38.8% (34.4% to 43.4%)
Portugal	3.3% (2.1% to 5.0%)	5.4% (3.9% to 7.6%)	64.7% (61.0% to 68.3%)	61.4% (57.7% to 65.1%)
Romania	11.5% (9.1% to 14.4%)	13.5% (11.0% to 16.5%)	37.3% (33.2% to 41.5%)	42.5% (38.3% to 46.9%)
Slovakia	4.3% (2.7% to 6.9%)	7.2% (5.0% to 10.1%)	61.8% (57.2% to 66.1%)	54.3% (49.7% to 58.8%)
Slovenia	0.1% (0.0% to 1.0%)	9.0% (6.6% to 12.3%)	85.0% (81.5% to 87.9%)	70.9% (66.5% to 74.9%)
Spain	0.0% (0.0% to 0.0%)	7.3% (5.3% to 9.9%)	66.6% (62.3% to 70.5%)	60.2% (55.8% to 64.4%)
Sweden	0.1% (0.0% to 1.0%)	8.9% (5.9% to 13.2%)	79.4% (74.5% to 83.5%)	75.3% (69.8% to 80.2%)
EU	2.0% (1.6% to 2.4%)	7.1% (6.5% to 7.9%)	58.8% (57.4% to 60.1%)	51.1% (49.7% to 52.4%)

HTP = heated tobacco product.

Appealing: Do you find HTPs appealing?

Effective aid for smoking cessation: Do you think that the use of HTPs helps tobacco smokers quit?

Regulate as strictly as cigarettes: Do you think that HTPs should be regulated as strictly as cigarettes?

Stricter point-of-sale advertising restrictions: Would you be in favor of keeping HTPs out of sight in shops or points of sale?

In fully adjusted regression models, women were less likely than men to perceive HTPs as effective aids for smoking cessation (PR = 0.82, 0.73 to 0.91). Younger individuals (ages 15–24 and 25–39) were significantly more likely than those aged ≥55 to find HTPs appealing (PR = 1.88, 1.19 to 2.95 and PR = 1.54, 1.04 to 2.28, respectively) and to perceive them as effective for smoking cessation (PR = 2.29, 1.58 to 3.33 and PR = 1.52, 1.15 to 2.02, respectively). Financial difficulty was associated with reduced support for regulating HTPs as strictly as cigarettes (PR = 0.89, 0.85 to 0.93) and for point-of-sale restrictions (PR = 0.93, 0.88 to 0.98). Conversely, individuals who had completed their education at age 20 or older were more likely to support both stricter regulation of HTPs (PR = 1.16, 1.08 to 1.25) and point-of-sale restrictions (PR = 1.14, 1.07 to 1.22) compared to those with lower education levels (0–15 years). Living with children was associated with increased support for stricter regulation of HTPs (PR = 1.06, 1.02 to 1.11) and point-of-sale advertising restrictions (PR = 1.05, 1.01 to 1.09). Meanwhile, compared to those employed, students, house persons, and retirees showed greater support for stricter regulatory measures and for keeping HTPs out of sight at points of sale. However, these groups were less likely to perceive HTPs as effective aids for smoking cessation (Table 4).

**Table 4:** Multi-level Poisson Regression Estimating Associations Between Sociodemographic Factors and Perceptions Related to HTPs Among Never Users of Tobacco, e-Cigarettes, or HTPs Across the EU

Variables	Appealing	Effective aid for smoking cessation	Regulate as strictly as cigarettes	Stricter point-of-sale advertising restrictions
	Prevalence ratio (95% confidence interval)			
Gender				
Male (Ref.)	1	1	1	1
Female	0.80 (0.64 to 1.01)	0.82 (0.73 to 0.91)	1.02 (0.99 to 1.06)	1.04 (1.00 to 1.07)
Age (years)				
≥55 (Ref.)	1	1	1	1
15–24	1.88 (1.19 to 2.95)	2.29 (1.58 to 3.33)	1.00 (0.91 to 1.10)	0.98 (0.88 to 1.09)
25–39	1.54 (1.04 to 2.28)	1.52 (1.15 to 2.02)	0.99 (0.94 to 1.04)	0.98 (0.92 to 1.04)
40–54	0.91 (0.53 to 1.58)	1.15 (0.92 to 1.43)	1.02 (0.99 to 1.05)	1.00 (0.96 to 1.04)
Difficulty paying bills				
Almost never/never (Ref.)	1	1	1	1
From time to time/most of the time	1.03 (0.73 to 1.46)	1.14 (0.97 to 1.34)	0.89 (0.85 to 0.93)	0.93 (0.88 to 0.98)
Community type				
Rural (Ref.)	1	1	1	1
Urban	1.06 (0.81 to 1.38)	1.08 (0.88 to 1.33)	1.02 (0.96 to 1.07)	1.01 (0.95 to 1.07)
Education (age at completion)				
0–15 years (Ref.)	1	1	1	1
16–19 years	1.19 (0.71 to 2.01)	1.12 (0.84 to 1.50)	1.06 (0.98 to 1.15)	1.04 (0.98 to 1.11)
≥20 years	1.05 (0.61 to 1.81)	1.14 (0.82 to 1.57)	1.16 (1.08 to 1.25)	1.14 (1.07 to 1.22)
Still studying	2.08 (0.83 to 5.22)	1.12 (0.67 to 1.86)	1.07 (0.96 to 1.20)	1.02 (0.90 to 1.17)
Living with children				
No (Ref.)	1	1	1	1
Yes	1.38 (1.12 to 1.70)	1.07 (0.92 to 1.25)	1.06 (1.02 to 1.11)	1.05 (1.01 to 1.09)
Employment status				
Employed (Ref.)	1	1	1	1
Unemployed	0.29 (0.75 to 1.12)	1.29 (0.92 to 1.83)	1.04 (0.96 to 1.14)	1.02 (0.94 to 1.11)
Students/house persons/retired	0.76 (0.41 to 1.42)	0.74 (0.58 to 0.96)	1.11 (1.05 to 1.17)	1.10 (1.03 to 1.17)

HTP = heated tobacco product

Appealing: Do you find HTPs appealing?

Effective aid for smoking cessation: Do you think that the use of HTPs helps tobacco smokers quit?

Regulate as strictly as cigarettes: Do you think that HTPs should be regulated as strictly as cigarettes?

Stricter point-of-sale advertising restrictions: Would you be in favor of keeping HTPs out of sight in shops or points of sale?

## Discussion

This study highlights the varied perceptions of e-cigarettes and HTPs among never users of tobacco, e-cigarettes, or HTPs in the EU. Men and younger individuals were more likely to perceive these products as effective aids for smoking cessation. Support for stricter regulation and point-of-sale display restrictions was generally stronger among those living with children, as well as students, house persons, or retirees, while individuals facing financial difficulties were less supportive. These findings highlight the importance of considering sociodemographic factors when developing regulatory policies for emerging tobacco and nicotine products.

Although the EU introduced the TPD and later activated the clause for HTPs to strengthen the regulatory framework for emerging tobacco and nicotine products, overall policies vary across MSs, as each retains jurisdiction over key areas such as bans, domestic advertising, taxation, and flavor regulations.^22^^,^^23^ For example, countries like Finland have implemented regulations that exceed TPD requirements, including comprehensive bans on flavors and restrictions on marketing, display, and distance selling.^24^ In contrast, some MSs, such as the Czech Republic, have only partially implemented the TPD, leaving gaps in regulations like packaging and health warnings.^25^ These discrepancies in national regulations likely influence perceptions, particularly regarding the appeal and perceived effectiveness of e-cigarettes and HTPs as cessation tools.^26^

This is perhaps illustrated by the contrast between Malta, which has the highest percentage of respondents finding e-cigarettes appealing, and Latvia, which has the lowest. Malta does not require a specific retail license for e-cigarette sales, and domestic sales via the internet are not regulated. In contrast, Latvia has fully banned online sales and banned e-cigarette sales via vending machines, along with imposing restrictions on their use in public spaces.^27^ Notably, Romania reported the highest prevalence of HTP appeal. Romania has been one of the key European markets for HTPs and has been identified as one of the EU MSs with the highest levels of HTP advertising.^28^

Similarly, support for stricter regulatory measures and point-of-sale display restrictions varies across MSs. Remarkably, Estonia, which has the highest prevalence of e-cigarette use in the EU,^2^ demonstrates the highest public support for point-of-sale advertising restrictions. This could reflect the complex relationship between the prevalence of emerging tobacco and nicotine products and support for stricter restrictions. On one hand, it could be argued that high prevalence may lead to normalization of use and broader social acceptance. For instance, in some countries, attitudes toward e-cigarettes among health professionals may be more positive, even though they are not officially recommended as cessation tools. As such, the public may be more likely to perceive them as effective aids for quitting, extend this perception to other similar products, and consequently be less supportive of strict regulation. On the other hand, in regions where these products are less prevalent, never users may perceive them as less of a concern, resulting in lower support for restrictions,^29^ whereas higher prevalence may make never users perceive these products as more intrusive, driving stronger support for tobacco control policies.^30^

The overall appeal was low among never users, which aligns with previous studies in the United States, where individuals who had never used tobacco or nicotine products showed little interest in HTPs and modified risk tobacco products.^31^^,^^32^ Appeal was notably higher in some MSs, particularly among young people. Since individuals who find products appealing are at greater risk of future initiation, this increased appeal raises further concerns.^33^ Younger age groups are more likely to be exposed to emerging tobacco and nicotine product advertising, which may contribute to their glamorization, despite the uncertainty surrounding their mid- and long-term health impact.^34^^,^^35^ The design of these emerging products, often featuring appealing flavors to attract younger individuals and marketed as safer alternatives, shapes perceptions of harm.^36^ Stricter restrictions on marketing targeting vulnerable populations, particularly youth, could help prevent nicotine addiction and reduce the risk of e-cigarettes and HTPs serving as gateways to traditional cigarettes.^37^

Additionally, our findings align with a study from the United States showing that nicotine-naive individuals from lower socioeconomic backgrounds are more likely to misperceive the risks of the modified risk tobacco products.^32^ However, we found that nicotine-naive men were more likely to perceive e-cigarettes and HTPs as appealing, whereas a US study found that women were more likely than men to perceive flavored e-cigarettes as easier to use.^38^ Although our study did not differentiate between flavored and non-flavored products, available evidence suggests that perceptions of harm and appeal may vary depending on product characteristics. This emphasizes the need for more targeted public health interventions across EU MSs, particularly to protect vulnerable populations, mitigate the risk of nicotine addiction, and prevent initiation.

Although perceptions around the effectiveness of e-cigarettes and HTPs as smoking cessation aids are not necessarily associated with experimentation or initiation of use among never users, it’s a measure that provides additional insight into how people view them. Although HTPs have not been shown to help with smoking cessation,^13^ there is some evidence that supports the role of e-cigarettes in smoking cessation in certain contexts.^39^ However, it’s unclear if this perception may lead never users of nicotine to consider them less harmful and, hence, indirectly encourage experimentation. Interestingly, the highest percentage of respondents who perceived e-cigarettes and HTPs as effective smoking cessation aids was in Ireland, although appeal for both products was lower than the EU average. Even though they are not officially recommended as aids to quit smoking by the Irish healthcare system,^40^ e-cigarettes are popular among those trying to quit in Ireland,^2^ perhaps due to influences from the neighboring United Kingdom, where e-cigarettes are used as cessation aids in the National Health Service.^41^

Public support for or against regulations on the marketing and use of e-cigarettes and HTPs can significantly shape policy outcomes,^37^ as policymakers are more likely to implement measures with strong public backing. Notably, our study focuses on never users; support for regulation tends to be lower among current or former users.^42^ The tobacco control community in Europe can focus on population subgroups identified as less supportive in our analysis and advocate for the necessity and public health value of such measures. Increased public understanding and engagement, especially within these specific groups, could help foster broader support for policies aimed at protecting public health.

### Strengths and Limitations

This study employs a large, representative sample of 13 436 participants across 27 EU MSs and utilizes a standardized set of survey questions. The dataset is recent, ensuring the findings are relevant to current policy discussions. By focusing exclusively on never users of tobacco, e-cigarettes, or HTPs, we provide a unique perspective on perceptions of these products among a critical population subgroup. However, limitations exist, particularly regarding the interpretation of certain survey questions. For instance, the term “appealing” may be interpreted differently by respondents, potentially referring to factors such as style, taste, or other qualities. Additionally, the question “Do you think that e-cigarettes should be regulated as strictly as cigarettes?” is relatively vague and depends on the local regulatory context, which may lead to varying interpretations. Some individuals may consider strict regulation to encompass comprehensive restrictions on advertising, packaging, and sales similar to those imposed on traditional cigarettes, while others may interpret it as including complete bans on use in some public spaces. Some MSs have small sample sizes, which may reduce the power of subgroup analyses and limit the granularity of country-specific insights. Exclusion of responses from individuals identifying outside the binary gender categories included may limit inclusivity and the generalizability of findings to underrepresented groups. Moreover, the survey’s questions measure perceptions and support for regulations but do not capture nuanced individual experiences or motivations, such as the context of appeal or the factors driving support for regulatory measures. Furthermore, self-reported survey data may be subject to misclassification between e-cigarettes and HTPs. While interviewers provided verbal descriptions of each tobacco and nicotine product assessed at the beginning of the survey, the absence of accompanying images may have caused some confusion. Similarities in device design, along with the more established market presence of e-cigarettes across EU MSs, may have led respondents to inadvertently report use of one product type as the other. However, this is unlikely to have resulted in substantial misclassification. These limitations may result in an underestimation or oversimplification of sociodemographic disparities and cross-national differences.

## Conclusion

In conclusion, the significant variation in perceptions of e-cigarettes and HTPs across EU MSs, particularly among young people, highlights the critical need for more tailored and comprehensive legislation and implementation in regulating these products. As demonstrated by the public support for regulatory measures in certain MS, the scope of the TPD could be expanded to consider the diverse perceptions of emerging tobacco and nicotine products and their potential appeal to never users, particularly young people. Policymakers may consider enhancing restrictions on marketing and display while tailoring interventions to address the needs of specific sociodemographic subgroups. An updated version of the TPD is required to address these emerging trends and ensure effective regulation that protects public health.

## Supplementary Material

Ecigs_and_HTP_perceptions_NTR_R1_Clean_ntaf168

## Data Availability

Data not currently publicly available.
