# Supplementary material for: Perceptions of e-Cigarettes and Heated Tobacco Products Among Never Users of Nicotine in the European Union
Source: Nicotine Tob Res. 2025 Aug 1;27(12):2223–30. doi: 10.1093/ntr/ntaf168 (PMC12641166; doi:10.1093/ntr/ntaf168)
Supplement: Ecigs_and_HTP_perceptions_NTR_R1_Clean_ntaf168 [file ecigs_and_htp_perceptions_ntr_r1_clean_ntaf168.docx]

Suppl. Table 1. Descriptive demographics (n=13,436)

| Variables | *N* | Weighted Proportion |
| --- | --- | --- |
| **Gender** | | |
| Male | 5,110 | 41.9% |
| Female | 8,304 | 58.1% |
| **Age (years)** | | |
| 15-24 | 1,314 | 13.5% |
| 25-39 | 2,492 | 21.1% |
| 40-54 | 3,150 | 23.7% |
| 55+ | 6,478 | 41.6% |
| **Difficulty paying bills** | | |
| Almost never/never | 9,189 | 68.9% |
| From time to time/most of the time | 4,148 | 31.1% |
| **Community type** | | |
| Rural | 4,704 | 32.4% |
| Urban | 8,729 | 67.6% |
| **Education (age at completion)** | | |
| 0-15 years | 1,647 | 14.7% |
| 16-19 years | 5,239 | 39.7% |
| 20+ years | 5,204 | 33.7% |
| Still studying | 1,158 | 11.9% |
| **Living with children** | | |
| Yes | 4,247 | 31.2% |
| **Employment status** | | |
| Employed | 6,994 | 52.7% |
| Unemployed | 399 | 3.3% |
| Students/house persons/retired | 6,043 | 44.0% |
| **E-cigarettes appealing** | | |
| Yes | 388 | 2.6% |
| **HTPs appealing** | | |
| Yes | 276 | 2.0% |
| **E-cigarettes** **effective aid for smoking cessation** | | |
| Yes | 1,446 | 11.2% |
| **HTPs** **effective aid for smoking cessation** | | |
| Yes | 1,009 | 7.2% |
| **E-cigarettes** **regulate as strictly as cigarettes** | | |
| Yes | 8,610 | 58.4% |
| **HTPs regulate as strictly as cigarettes** | | |
| Yes | 8,654 | 58.8% |
| **Stricter point-of-sale advertising restrictions (e-cigarettes)** | | |
| In favour | 7,868 | 50.8% |
| **Stricter point-of-sale advertising restrictions (HTPs)** | | |
| In favour | 7,887 | 51.1% |
| ***N*** | 13,436 |  |

HTP: Heated tobacco product

E-cigarettes appealing: Do you find e-cigarettes appealing?

HTPs Appealing: Do you find HTPs appealing?

E-cigarettes effective aid for smoking cessation: Do you think that the use of e-cigarettes helps tobacco smokers quit?

HTPs effective aid for smoking cessation: Do you think that the use of HTPs helps tobacco smokers quit?

E-cigarettes regulate as strictly as cigarettes: Do you think that e-cigarettes should be regulated as strictly as cigarettes?

HTPs regulate as strictly as cigarettes: Do you think that HTPs should be regulated as strictly as cigarettes?

Stricter point-of-sale advertising restrictions (e-cigarettes): Would you be in favour of keeping e-cigarettes out of sight in shops or points of sale?

Stricter point-of-sale advertising restrictions (HTPs): Would you be in favour of keeping HTPs out of sight in shops or points of sale?

**Suppl. Table 2. Distribution of missing responses across all variables (n=13,436)**

| Variables | *N* | Weighted Proportion |
| --- | --- | --- |
| **Gender** | | |
| Missing | 22 | 0.08% |
| **Age (years)** | | |
| Missing | 2 | 0.02% |
| **Difficulty paying bills** | | |
| Missing | 99 | 1.0% |
| **Community type** | | |
| Missing | 3 | 0.01% |
| **Education (age at completion)** | | |
| Missing | 188 | 1.5% |
| **Living with children** | | |
| Missing | 85 | 0.5% |
| **Employment status** | | |
| Missing | 0 | 0.0% |
| **E-cigarettes appealing** | | |
| Missing | 0 | 0.0% |
| **HTPs appealing** | | |
| Missing | 0 | 0.0% |
| **E-cigarettes** **effective aid for smoking cessation** | | |
| Missing | 0 | 0.0% |
| **HTPs** **effective aid for smoking cessation** | | |
| Missing | 0 | 0.0% |
| **E-cigarettes** **regulate as strictly as cigarettes** | | |
| Missing | 0 | 0.0% |
| **HTPs regulate as strictly as cigarettes** | | |
| Missing | 0 | 0.0% |
| **Stricter point-of-sale advertising restrictions (e-cigarettes)** | | |
| Missing | 0 | 0.0% |
| **Stricter point-of-sale advertising restrictions (HTPs)** | | |
| Missing | 0 | 0.0% |

HTP: Heated tobacco product

E-cigarettes appealing: Do you find e-cigarettes appealing?

HTPs Appealing: Do you find HTPs appealing?

E-cigarettes effective aid for smoking cessation: Do you think that the use of e-cigarettes helps tobacco smokers quit?

HTPs effective aid for smoking cessation: Do you think that the use of HTPs helps tobacco smokers quit?

E-cigarettes regulate as strictly as cigarettes: Do you think that e-cigarettes should be regulated as strictly as cigarettes?

HTPs regulate as strictly as cigarettes: Do you think that HTPs should be regulated as strictly as cigarettes?

Stricter point-of-sale advertising restrictions (e-cigarettes): Would you be in favour of keeping e-cigarettes out of sight in shops or points of sale?

Stricter point-of-sale advertising restrictions (HTPs): Would you be in favour of keeping HTPs out of sight in shops or points of sale?
